# Nutrition economics: towards comprehensive understanding of the benefits of nutrition

**DOI:** 10.3402/mehd.v23i0.18585

**Published:** 2012-06-18

**Authors:** Aki Koponen, Mari Sandell, Seppo Salminen, Irene Lenoir-Wijnkoop

**Affiliations:** 1 Centre for Collaborative Research, Turku School of Economics, University of Turku, Turku, Finland; 2 Functional Foods Forum, University of Turku, Turku, Finland; 3 Departement of Pharmaceutical Sciences, Utrecht University, Utrecht, The Netherlands

**Keywords:** Nutrition, economics, public health, effectiveness, product development, service networks

## Abstract

There has been an increase in the knowledge and interest on nutrition, and functional foods have gained popularity over the last few decades, and the trend is increasing. Probiotics and prebiotics are among the most studied functional foods. Nutrition economics has been defined as the discipline dedicated to researching and characterising health and economic outcomes in nutrition for the benefit of society. The concept and its application to probiotics and prebiotics will be discussed in terms of health and economic benefits and their evaluation. Health economics and concrete applications showing how to maximise long-term nutritional benefits will contribute to motivate consumers in making food choices based on a rational understanding of their own interest. We present a model that shows that nutrition economics can be used as an analytical tool for product and service network development.

The interaction of foods, nutrition, and public health is increasingly recognised as a key element of health and wellbeing and also a factor in quality of life. Scientific evidence is gradually unravelling the link between food and health maintenance as well as the potential of food to prevent or delay disease development.

In this context, functional foods have gained popularity over the last few decades, and the trend is increasing. Functional foods, which are found in virtually all food categories, can be described as foods that provide more than simple nutrition and that supply additional physiological benefit to the consumer (1). A substantial international market exists for functional foods both in terms of market value and variety of premium products.

Currently, in the field of health care, the economic assessment has rapidly developed, emerging from the increasing pressure on health care budgets and the growing interest in cost-effective and evidence-based health care. This has resulted in an increasing number of cost-effectiveness studies being completed – in fact a particular research discipline, called health economics, has evolved – and a growing role of health-economic arguments in policy making.

However, the fields of functional foods and health economics have only recently been interconnected (2), and reliable data on cost-effectiveness or cost benefits of nutritional interventions for health management are often lacking. The medical profession, in cooperation with the pharmaceutical industry and the hospital community, has introduced science-based economic evaluation methods for the assessment of health management programs. In this area, also standardised treatment protocols, in particular in the hospital setting, have been introduced and assessed. This has allowed to establish the health-economic principles of cost-benefit and cost-effectiveness assessments. Until now, however, no specific approach has been established for estimating the potential of food in improving the allocation of the available health care resources, in spite of a clear need by both policymakers and health professionals, as well as consumers. A recent report by the World Bank states that cost-effectiveness of functional foods in reducing disease burden and lost productivity is an important research gap (3), even though ‘the popularity of functional foods is increasing and the effect on the food industry is evident’.

The objective of this work is to propose a schema that sketches how nutrition economics can be used to emphasise the relevance of nutritional benefits, to provide a framework for nutrition guidance and a tool for designing new foods in a perspective of personalised nutrition advice as a future procedure for individuals, companies, and societal structures. Examples will be taken from the field of probiotics and prebiotics as these are among the most studied foods for health benefits (11). The WHO expert group definition of probiotics states that probiotics are ‘live microorganisms which when administered in adequate amounts confer a health benefit on the host’ (4). The prebiotic definition according to FAO states that: ‘A prebiotic is a non-viable food component that confers a health benefit on the host associated with modulation of the microbiota’ (5).

Although the functional properties and healthiness of probiotics and prebiotics will increase the nutritional quality and health benefits of a food product, consumers are different in their food choices motives (6). The critical sensory properties contributing to liking of product may be very different for consumers considering health and ethical concern as important motives, compared to consumers considering convenience, mood, and familiarity as important. Liking again is a crucial factor in enjoyability on food markets. This type of knowledge increases the challenges for development of functional food and also nutritional education.

This paper presents a novel framework of economic analysis where more traditional health economics methods are linked to product development and service network creation. The framework introduces the idea of a positive feedback system between economic analysis of nutrition and product and service network development. That is, business development can benefit from economic analysis of nutrition, and simultaneously service network is a crucial element for wider realisation of system level benefits of nutrition.

## Complexity of food choices

Food choice is often a very individual decision. Food products contain many kind of sensory and non-sensory factors that may have an influence on the individual choice. Sensory factors include experiences perceived with both chemical and physical senses. Nutritional information, health claims, together with price, origin, and brand may play an important role to motivate consumers in making their choices. In addition, the consumer-related personal factors such as genetic variation, age, gender, tradition and values, attitudes, and demographic and socioeconomic status have a complex effect on the degree of pleasantness and acceptability and moreover to food perception. We live effectively in our personal sensory worlds. Based on the economic science definition of selecting foods, individual consumers may choose food items based on rational understanding of maximising long-term benefits for themselves.

Understanding the individual indicators is the key point leading us to next generation and personalised product development. More information is clearly needed for the decision making and wider efforts should be placed by the food manufacturers to develop healthy and tasty choices to promote good balanced and sustainable nutrition. In a similar manner, food service providers and nutrition professionals should propose services, which will successfully result in cost savings at varying levels – for the individual, for health care, and for the whole society. These factors should be thoroughly assessed not only to take into account the expenses of societal programmes needed to induce a change in behaviour but also especially in terms of the rising costs of so-called lifestyle diseases and stress symptoms disturbing wellbeing, which are increasing in prevalence. These include among others diabetes, osteoporosis, obesity, and cancer as well as the still high level of cardiovascular diseases. The balance for dietary prevention and treatment costs against health care costs and use of pharmaceutical preparations needs to be more clearly identified and cost benefits evaluated for each step in risk reduction, prevention, and treatment.

## Economic aspects of nutritional benefits

Nutrition economics has been defined as the discipline dedicated to researching and characterising health and economic outcomes in nutrition for the benefit of society. Within the setting of a production-driven context, cost-effect calculations will often take other aspects into consideration, such as environmental issues and the use of natural resources. Hence, the correction mechanisms of possible positive or negative externalities add a complementary dimension, which will be discussed below based on the following three items:

1. Maintaining health by appropriate nutrition (*Better health equity for different socioeconomic classes, improved productivity, and reduced health care costs*);
2. Alleviating symptoms and reducing risk of disease by nutrition (*Reduction of absenteeism from work, optimisation of resource utilisation by reduced hospital stays, and more focus on those patients who need attention*);
3. Improving wellbeing and quality of life by nutrition (*increased mobility and access to services, enhanced service utilisation*).

All the above-mentioned themes may at times also apply to probiotic and prebiotics. The first theme is actually the simplest one and requires only the application of nutrition recommendations. In the food patterns of the general population, this would concern the contribution of probiotics and prebiotics to a healthy condition through their interaction with the gut microbiota. Most investigators conclude that results are promising; nevertheless, more research on the causality between products and effects as well as on the extent of the impact is needed to provide concrete estimations about the efficiency.

The second effect of nutrition might often be comparable to the one of health care, decision of a treatment can be based on cost savings (or other measures of pharmacoeconomics). A good example here of can be found in interventions trials that study the impact of probiotics on diarrhoea and in particular the case of acute paediatric diarrhoea (7) and antibiotic-associated diarrhoea (AAD) (8) The incidence of AAD varies with the class of antibiotic used and with the characteristics of patients treated: AAD has been observed in a wide variety of patient populations including orthopaedic, obstetric/gynaecologic, intensive-care-unit patients, as well as ambulatory patients in the outpatient setting. One of the mechanisms by which antibiotics cause diarrhoea is by alteration of the commensal gut microflora. In such cases, a medical decision or recommendation to use probiotics aims at minimising the deleterious impact of antibiotics on the gastrointestinal flora. Currently, several initiatives are looking more closely into the health-economic consequences of these strategies and their potential to reduce health care expenditures.

In the case of prevention, the second approach becomes more complex, since the cost-effectiveness of preventive care is often difficult to measure, but on the other hand, assessment of symptom alleviation can be easier. An interesting possibility in this context is the use of modellisation techniques, as illustrated by a pilot analysis that assessed the cost-effectiveness of the use of prebiotics for the primary prevention of atopic dermatitis in an at-risk population (10). Atopic dermatitis is the most common inflammatory skin disease in children, affecting 10–15% of children in the developed world and, increasingly, those in the developing world. This study showed that the favourable health benefit associated with the use of prebiotics results in positive short- and long-term health economic outcomes.

**Fig. 1:**
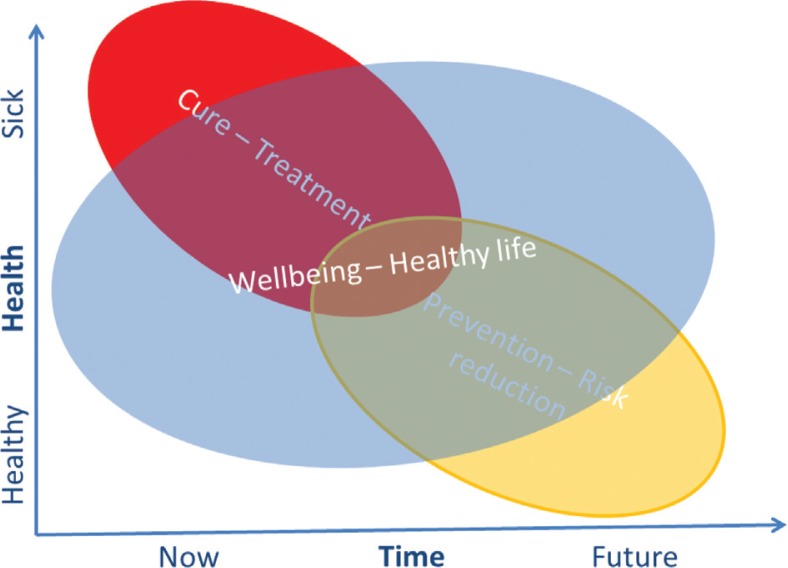
Focus of economic analysis of nutrition with respect to health status and realisation of the outcome.

The main differences between both are the health status of the user and realisation of the assumed benefit of the nutrition. In the case of an existing symptom, the relief can be observed more or less rapidly, while in the second case, a healthy person reduces his/her risk of disease somewhere in the future. This is presented in Fig. 1.

These themes are closely linked to the traditional health economics and methods of health economics are more or less straightforwardly applicable [for requirements of viable analysis, see (9)]. The third theme is the most challenging since general wellbeing is not very simply measurable, i.e. individual utility functions are neither very simple nor well-identified. Respectively, the effects are not very clear, and typically, there are plenty of interactions between different factors having an effect on individual wellbeing. Since effects are also often under dispute, it is justified to say that consumers having individual preference do make very much decisions under uncertainty. The consumer then knows the effect of the diet only after having tried it. If the response time is short, we can talk about experience goods in economic terms, but in the case of very long-term risk reduction type of goal, it is possible that customer never knows for sure if the diet worked. In the latter case, the product or service is called credence good. As the manufacturer producing the food has more information on the health functionality of the product than the consumer can readily verify, this can lead to asymmetric information. As a result, the ability of the functional food industry to credibly communicate will play a critical role in the marketplace success and subsequent health benefits in the population. Positive government regulations regarding heath claims and food labelling, such as those that allow food containing plant sterols to claim CHD prevention, would obviously go a long way towards ameliorating information asymmetry concerns by giving consumers as well as health care providers an impartial source of information on the credence aspects of functional foods.

**Fig. 2:**
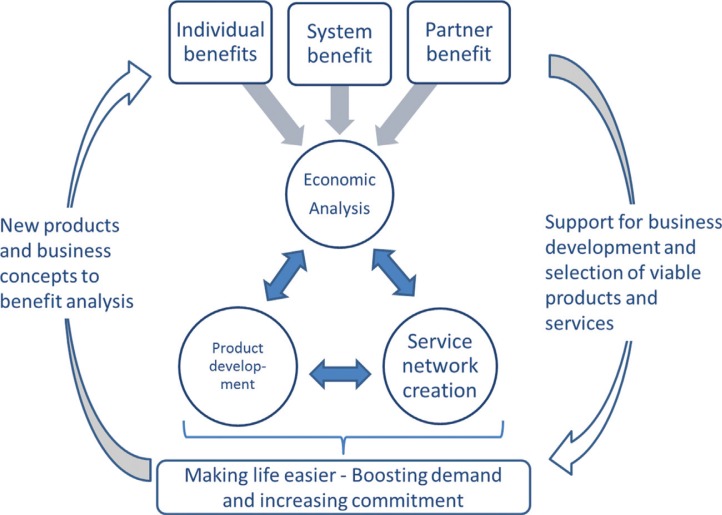
Economic interplay of nutrition, product development & service networks.

The level of uncertainty and limited ability to identify the effects leads to the approach of this paper. That is, we see the market as a discovery process and in this endeavour, there should be different kind of actors, enabling not only supply and demand to exist but also to make them meet. Fig. 2 illustrates the system consisting of the economic interplay of nutrition, product development, and service (or value) network creation. The role of the economic analysis is twofold. Firstly, economic analysis increases the knowledge about the effectiveness of the certain product, diet, or nutrition intervention. This knowledge can be used when marketing the product for the customers (individual benefits – how consumer's wellbeing improves by using the product), applying for public support (system benefits – how consuming the product reduces health care costs), or gathering partners for larger scale supply (partner benefits – how promoting a product helps partners to fulfil their value proposition to their customers). By service network, either a healthier diet or even a simple effective product can be made familiar to more consumers. Interaction between service provider and consumer both makes the consumer more committed to using products with positive health effects and also allows to collect information about the needed product development. Hence, the service network both boosts the demand of original products and safeguards the product evolution.

This rather abstract conceptualisation underlines the importance of nutrition economics. That is, health economics and concrete applications showing how to maximise long-term nutritional benefits will contribute to motivate consumers in making food choices based on a rational understanding of their own interest. The model also shows that nutrition economics can be used too as an analytical tool for product and service network development.

## Conclusion

A general methodology for nutrition economics is urgently needed for the background estimation and to provide tools for individual consumers, food manufacturers, food service operators, health care systems, and nutrition and health professionals. A societal impact of dietary changes should also be assessed based on costs and savings for the community as a whole.

We present a novel framework of economic analysis where more traditional health economic methods are linked to product development and service network creation. The framework introduces concepts with a positive feedback system between economic analysis of nutrition and product and service network development. Thus, business development can benefit from economic analysis of nutrition and simultaneously service network is a crucial element for wider realisation of system level benefits of nutrition for individual consumers and the society.
